# Supplementary material for: On the osmotic pressure of cells
Source: QRB Discov. 2022 Jul 11;3:e12. doi: 10.1017/qrd.2022.3 (PMC10392628; doi:10.1017/qrd.2022.3)
Supplement: Supplementary file 1 [file S2633289222000035sup001.docx]

Supplementary Information:

On the osmotic pressure of cells

Håkan Wennerström, Division of Physical Chemistry, Department of Chemistry, Lund University, P.O Box 124, SE-22100 Lund, Sweden,

and

Mikael Oliveberg, Department of Biochemistry and Biophysics, Arrhenius Laboratories for Natural Sciences, Stockholm University, SE-106 91 Stockholm, Sweden.

*Quantitative estimate of the contribution to the intracellular osmotic pressure from the proteins’ translational and rotational degrees of freedom.* The vant Hoff equation for the osmotic pressure is valid for proteins in dilute solution. In this limit, the protein molecules are free to translate and rotate. However, for typical intracellular protein concentrations, both translation and rotation is affected by the interaction with the neighbouring protein molecules resulting in a decreased entropy. It is a demanding task to accurately determine the contribution to the osmotic pressure from this effect, even if the protein-protein pair interaction would be known. Here, we resort to an estimate of the effect using several approximations as follows. The protein-protein pair-interaction *V*_ij_ depends on the respective orientations $\Omega_{i}$ and $\Omega_{j}$, where $\Omega$omega denotes the three eulerian angles. We write the interaction as the sum of an isotropic distance dependent term *V*^i^ and an orientation dependent term *V*^o^

$V_{ij}\left( r_{ij},\Omega_{i},\Omega_{j} \right)=V_{ij}^{i}\left( r_{ij} \right)+V_{ij}^{o}\left( r_{ij},\Omega_{i},\Omega_{j} \right)$ (Eq. S1),

where it is assumed the proteins have a fixed conformation. The orientation dependent part is defined so that it has a value zero when averaged over the orientations of both molecules

$\iint V_{ij}^{o}\left( r_{ij},\Omega_{i},\Omega_{j} \right)d\Omega_{i}d\Omega_{j}=0$ (Eq. S2).

Now, we make the first approximation by assuming that the translational and orientational contributions can be considered as independent so that one can replace *r*_ij_ in *V*^o^_ij_ by its average value *r*_ij_^av^ at a given concentration. Then, as a second approximation, the contribution from the translation is estimated from the Carnahan Starling expression (N.F.Carnahan and K.E.Starling; (1969) J.Chem.Phys. 51, 635).

$\pi_{osm}=c_{p}kT\frac{1+\varphi+\varphi^{2}-\varphi^{3}}{(1-\varphi)^{3}}$ (Eq. S3),

which is valid for a hard sphere system, and where $\varphi$ is the volume fraction of proteins. For $\varphi$ = 0.35, the estimated contribution to the osmotic pressure is π ≈ 5 *kTc*_p_.

To estimate the rotational contribution, it is convenient to introduce a third approximation stating that the orientation dependence of *V*^o^ is independent of separation, so that

$V_{ij}^{o}=f_{r}(r_{ij})f_{\Omega}(\Omega_{i},\Omega_{j})$ (Eq. S4).

This would, for example, be valid for a single component of a multipole expansion of the interaction. The orientational contribution to the osmotic pressure is obtained from the derivative of the orientational free energy with respect to volume and

$\frac{dA_{rot}}{dV}=\frac{dA_{rot}}{dc}\frac{dc}{dV}\approx\frac{dA_{rot}}{dr_{av}}\frac{dr_{av}}{dc}\frac{dc}{dV}=\frac{1}{3}\frac{dA_{rot}}{dr_{av}}\frac{r_{av}}{V}$ (Eq. S5).

The rotational free energy is obtained from the logarithm of the rotational configurational integral

$Z_{rot}=\iiint exp(-V_{rot}/kT)\left\{ d\Omega_{i} \right\}_{1}^{N}$ (Eq. S6).

According to Eq. S4 V_rot_ can be written as

$V_{rot}=f_{r}(r_{av})\sum_{i>j} f_{\Omega}(\Omega_{i},\Omega_{j})$ (Eq. S7),

where the sum is over near neighbours. The derivative of the configurational integral is

$\frac{dZ_{rot}}{dr_{av}}=(-\frac{1}{kT})\iiint\frac{dV_{rot}}{dr_{av}}exp(-V_{rot}/kT)\left\{ d\Omega_{i} \right\}_{1}^{N}=\frac{1}{f_{r}(r_{av})}\frac{df_{r}}{dr_{av}}$ $\iiint V_{rot}exp(-V_{rot}/kT)\left\{ d\Omega_{i} \right\}_{1}^{N}=Z_{rot}\frac{1}{f_{r}(r_{av})}\frac{df_{r}}{dr_{av}}$ U_rot_  (Eq. S8).

So that using Eq. S5 and Eq. S8

$\pi_{rot}=\frac{c_{p}}{3}u_{rot}r_{av}\frac{dln(f_{r})}{dr_{av}}$ (Eq. S9).

Here, *u*_rot_ is the average rotational interaction energy of a protein molecule. For the case of a dipolar interaction this results in a contribution

$\pi_{rot}\approx\left( \frac{n}{4} \right)kTc_{p}$ (Eq. S10)

when there is *n* neighbours and the amplitude of *V*^0^_ij_ is 1 *kT*. Note that, in addition to the direct linear dependence on the protein concentration, there is an additional even stronger concentration dependence due to the effect on $f_{r}(r_{ij})$.

These estimates of the translational and rotational contributions to the osmotic pressure shows that there are large non-ideal effects giving a much higher osmotic pressure than what is estimated from Eq. 3. Taking the estimate of Eq. S10, the rotational contribution, using *n* = 10 near neighbours and a protein concentration of 15 mM, the resulting combined contribution to the osmolarity is 7.5 x 15 ≈ 110 mM. Although this is a rough estimate, it shows that the proteins contribute substantially to the intracellular osmotic pressure. The contribution is strongly concentration dependent and considering the uncertainties involved in this estimate we arrive at a range for the effect from 75 mM to 150 mM.
